# Acupressure versus parecoxib sodium in acute renal colic: A prospective cohort study

**DOI:** 10.3389/fmed.2022.968433

**Published:** 2023-01-09

**Authors:** Chiwei Chen, Zhenpeng Zhang, Mandi Lin, Zhigang Wang, Hao Liu, Hao Meng, Jun Wang, Ming Chen, Songtao Xiang, Yunqiao Qiu, Hong Liu

**Affiliations:** ^1^The First Affiliated Hospital of Guangzhou University of Chinese Medicine, Guangzhou University of Chinese Medicine, Guangzhou, Guangdong, China; ^2^The Second Affiliated Hospital of Guangzhou University of Chinese Medicine, Guangdong Provincial Hospital of Chinese Medicine, Guangzhou University of Chinese Medicine, Guangzhou, Guangdong, China; ^3^The Second Clinical College of Guangzhou University of Chinese Medicine, Guangzhou, Guangdong, China; ^4^Shenzhen Hospital (Longgang), Beijing University of Traditional Chinese Medicine, Shenzhen, Guangdong, China

**Keywords:** acupressure, parecoxib sodium, renal colic, alternative therapy, NSAIDs

## Abstract

**Background:**

Here provides a complementary treatment, acupressure at the Qiu acupoint, a novel acupoint, which potentially alleviates renal colic.

**Materials and methods:**

90 patients were included in this study. Acupressure-group patients (*n* = 46) were administered acupressure at the Qiu acupoint following a preset protocol. Parecoxib sodium-group patients (*n* = 44) were administered parecoxib sodium (40 mg) (*via* the direct intravenous route). The visual analog scale (VAS) was used to evaluate pain intensity at baseline and at 1, 5, 10, 20, 30, and 120 min after initiating the intervention. Linear mixed effects model was performed to detect the rate of decrease of VAS per time and their covariant effect on the efficacy of acupressure.

**Results:**

No significant statistical differences in baseline data and VAS scores were observed. The acupressure group obtained lower VAS scores at the 1st, 5th, 10th, and 20th minute than the parecoxib sodium group after initiating the intervention (mean: 4.33 vs. 7.61, mean difference (MD): 3.29, 95% CI: 0.23, 2.84; mean: 2.65 vs. 7.61, MD: 4.96, 95% CI: 4.44, 5.49; mean: 1.63 vs. 6.59, MD: 4.96, 95% CI: 4.48, 5.44; mean: 1.26 vs. 3.64 MD: 2.38, 95% CI: 1.87, 2.88; *P* < 0.05). The markedly effective rate was similar between the two groups. The linear mixed effects model demonstrated that acupressure at the Qiu point was significantly faster than parecoxib sodium in decreasing VAS scores with an estimate of –2.05 (95% CI: –2.51, –1.59, *p* = 0.000), especially within 10 minutes with an estimate of 0.18 (95% CI: 0.12, 0.25, *p* = 0.000).

**Conclusion:**

Acupressure at the Qiu acupoint is significantly faster than parecoxib sodium in decreasing VAS scores within 10 minutes.

**Clinical trial registration:**

http://www.chictr.org.cn/, identifier 2100047168.

## 1. Introduction

Acute renal colic is a common symptom presented to the emergency and urology departments, with lifetime risks of 12 and 6% in men and women, respectively (1). Among patients with acute abdominal pain, 31.18% of the cases are caused by acute renal colic (2). Acute renal colic reportedly affects 5–15% of the UK population, often requiring emergent administration (3). Non-steroidal anti-inflammatory drugs (NSAIDs) and opioids are the most common pharmacotherapies for acute renal colic (4). NSAIDs have proven to be effective drugs for acute renal colic and have been recommended by the European Association of Urology guidelines (5). However, the adverse and financial burdens posed by these painkillers should be taken into consideration. NSAIDs are associated with approximately 30% of hospital admissions for drug-related adverse events (6). In particular, older patients are vulnerable to NSAID side effects, such as gastrointestinal toxicity, cardiovascular adverse effects, and nephrotoxicity (7). Furthermore, healthy financial data generated in Australia demonstrated that opioid prescriptions are expected to reach approximately three million by 2030, thus raising Australian healthcare system costs from AUD$ 25.2 million to AUD$ 72.4 million (8). Hence, there is an urgent need to identify an alternative treatment with reduced painkiller-induced side effects and financial burdens.

Although acupressure has been proven to be effective in patients with pain caused by cancers, premenstrual syndrome, and labor (9–11), there is limited evidence corroborating the utility of acupressure for acute renal colic. Herein, we propose a novel alternative treatment, namely, acupressure at the Qiu acupoint. The Qiu acupoint was discovered by YQ, a urologist at the First Affiliated Hospital of Guangzhou University of Chinese Medicine, during his extensive clinical practice in treating acute renal colic for decades. The location of the Qiu acupoint is described in detail in the Section “2 Materials and methods.” Our previous clinical observations revealed that acupressure at the Qiu acupoint is advantageous (12). However, strong evidence is still lacking; hence, we performed a prospective cohort study on acupressure at the Qiu acupoint for acute renal colic.

## 2. Materials and methods

### 2.1. Study design

This prospective cohort study was conducted at the First Affiliated Hospital of Guangzhou University of Chinese Medicine between June and October 2021. This trial was registered at the Chinese Clinical Trial Registry ChiCTR (ChiCTR2100047168) and approved by the Ethics Committee of the First Affiliated Hospital of Guangzhou University of Chinese Medicine (ethical approval number: NO. ZYYECK【2020】083). This study was performed and drafted according to the STROBE statement.

### 2.2. Enrollment criteria

Patients were enrolled if they fulfilled the following inclusion criteria: (1) sudden lumbar and abdominal pain; (2) accompanying percussion pain of the kidney region’s affected side; (3) paleness, weakness, nausea, vomiting, and sweating accompanying pain; (4) perineal radiation pain; (5) lower urinary tract symptoms (frequent urination, urgent urination, and pain); (6) anal-irritation symptoms; and (7) microscopic or naked-eye hematuria. Patients who provided consent to participate were included in the study, and their ages ranged from 18 to 65 years.

### 2.3. Exclusion criteria

Patients with the following conditions were excluded: (1) lumbar and abdominal pain not calculous-related; (2) unconsciousness, mental disease, complications with other serious diseases or unstable vital signs, or inability to cooperate; (3) pregnancy, preparation for pregnancy, or lactation; (4) lumbar skin damage, ulceration, or severe urinary tract infection; (5) allergy to NSAIDs or previous history of gastrointestinal bleeding, active gastric ulcer, or related contraindications prohibited in drug instructions; and (6) history of taking oral calcium blockers and α-receptor blockers within 4 days before inclusion in the study.

### 2.4. Location of Qiu acupoint

**FIGURE 1:**
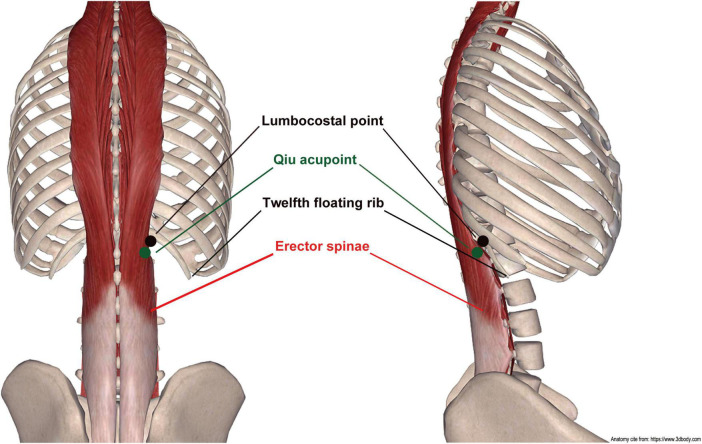
Anatomical location of Qiu acupoint (26).

The Qiu acupoint lies one thumb width (body size equal to 1.3 inches) down and one thumb width inside the lumbocostal point. The lumbocostal point is an intersection point crossed by the twelfth floating rib and erector spinae. Herein, we describe a simple method to find the Qiu acupoint. The lumbocostal point was pressed with one thumb, and the other thumb was subsequently pressed on the erector spinae in a position directly facing the thumb that pressed the lumbocostal point. Thereafter, the thumb that pressed on the erector spinae was moved down a distance of one thumb width (body size equal to 1.3 inches) along with the erector spinae, and the position of the thumb was thus above the Qiu point. The location of the Qiu acupoint is shown in Figure 1 and Supplementary Video.

### 2.5. Exposure

Patients in the acupressure group were administered acupressure at the Qiu acupoint. The detailed protocol is as follows: (1) The patient was placed in a lateral position with the painful side facing upward. (2) The Qiu acupoint was pressed in a force direction of 45° oblique to the spine with the thumb. (3) The power of the thumb was gradually increased for 1 min. The correct sign was the sensation of soreness and distention in the patient. Patients in the parecoxib sodium group were administered parecoxib sodium 40 mg resuspended in 20 ml 0.9% sodium chloride *via* the direct intravenous route.

### 2.6. Outcomes

The primary outcome was pain intensity, measured using a visual analog scale (VAS) that was 100-mm linear. VAS score ranges from 0 to 10 and every 10-mm represented 1 score. A score of 0 means no pain, and a score of 10 means the most excruciating pain. The VAS score was recorded by the investigator before the intervention and at 1, 5, 10, 20, 30, and 120 min after the intervention. The secondary outcomes were (1) onset time, defined as the time taken to achieve a 50% decrease in VAS score, and (2) the markedly effective rate, defined as the proportion of patients achieving a >60% VAS-score decrease within 30 min relative to each patient’s baseline. Adverse events were recorded at treatment initiation and until the patient was discharged from the hospital. Demographic and clinical data were collected from all the patients.

### 2.7. Propensity score matching

To adjust the potential confounding factors and baseline. PSM analysis was conducted to reduce these biases, PSM was conducted by creating a new control group from original control group in a way of selecting better matched subjects depending on a similar probability to receive the treatment. To achieve this, matching variables including gender, age, pain site which is defined as left side or right side (detected by ultrasound or X-ray), stone location which is defined as renal pelvis and upper ureter, middle ureter and lower ureter, and stone size which is defined as size <6 mm, 6 mm ≤ size <10 mm, and size ≥1 mm (detected by ultrasound or X-ray) were obtained as independent variables, and groups (control or treatment) were obtained as dependent variables. Those data were analyzed by logistic regression to find out the correlation of each variable with the selected treatment (13). The tolerance in PSM was 0.05 according to the previous report (14).

### 2.8. Statistical analysis

Variables are expressed as the mean ± standard deviation or values with 95% confidence intervals (CIs). All statistical analyses were performed using SPSS (version 25.0; IBM, Armonk, NY, USA). Student’s *t*-test was used to compare the continuous variables in baseline of demographic and clinical characteristics between acupressure group and parecoxib sodium group, the chi-square test was used to compare the categorical variables in baseline of demographic and clinical characteristics, VAS decrease, and markedly effective rate in 30 min between acupressure group and parecoxib sodium group, and the linear mixed effects models were used to compare the rate of decrease of VAS within 10 min between acupressure group and parecoxib sodium group and their covariant effect on the efficacy of acupressure. Statistical significance was set at *P* < 0.05. No data were missed.

## 3. Results

### 3.1. Baseline information

**FIGURE 2:**
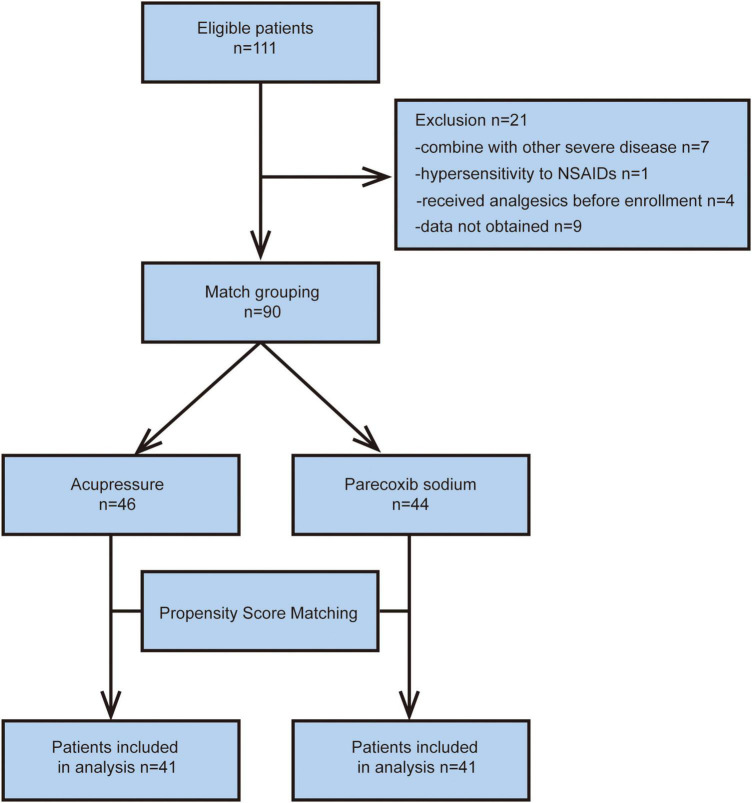
Flow chart of the study.

A total of 111 patients who met the inclusion criteria were included in the study. Twenty patients were excluded for the following reasons: (1) comorbid severe disease, (2) hypersensitivity to NSAIDs, (3) administration of analgesics before enrollment, and (4) refusal to participate. Ninety patients were divided into two cohorts. Forty-six patients were assigned to the acupressure group and 44 to the parecoxib sodium group. After PSM, both acupressure group and parecoxib sodium group were 41 patients. All patients in the two groups completed the study, and data were obtained for further analysis. Detailed information is shown in the flowchart in Figure 2. There were no statistical differences between the two groups in the following baseline characteristics: age, sex, pain site, stone location, and stone size. Details of the baseline characteristics are shown in Table 1.

**TABLE 1:** Baseline demographic and clinical characteristics of enumeration data.

Categorical variable^a^	Before PSM					After PSM				
	**Acupressure group (*n* = 46)**		**Parecoxib sodium group (*n* = 44)**		***P*-value**	**Acupressure group (*n* = 41)**		**Parecoxib sodium group (*n* = 41)**		***P*-value**
	**No.**	**%**	**No.**	**%**		**No.**	**%**	**No.**	**%**	
Age					0.941					1.000
≤39	13	28.3	11	25		10	24.4	10	24.4	
≥40, ≤59	31	67.4	31	70.5		29	70.7	29	70.7	
≥60	2	4.3	2	4.5		2	4.9	2	4.9	
Gender					0.361					0.821
Male	26	56.5	29	65.9		24	58.5	26	63.4	
Female	20	43.5	15	34.1		17	41.5	15	36.6	
Pain site					0.544					0.653
Left	28	60.9	24	54.5		26	63.4	23	56.1	
Right	18	39.1	20	45.5		15	36.6	18	43.9	
Stone location					0.774					0.856
Renal pelvis and upper ureter	18	39.1	18	40.9		16	39.0	17	41.5	
Middle ureter	10	21.7	7	15.9		9	22.0	7	17.1	
Lower ureter	18	39.1	19	43.2		16	39.0	17	41.5	
Stone size					0.834					1.000
<6 mm	7	15.2	6	13.6		6	14.6	6	14.6	
≥6 mm <10 mm	27	58.7	24	54.5		24	58.5	24	58.5	
≥ 10 mm	112	26.1	14	31.8		11	26.8	11	26.8	
**Continuous^b^ variable**	**Mean**	**SD**	**Mean**	**SD**	0.925	**Mean**	**SD**	**Mean**	**SD**	0.834
Age	45.5	9.0	45.6	9.2		46.1	8.5	45.7	9.4	

^a^ Categorical variables were analysis by *chi-square* tests.

^b^ Continuous variables were analysis by *t*-tests.

### 3.2. Effects on VAS-decrease rate and markedly effective rate

**FIGURE 3:**
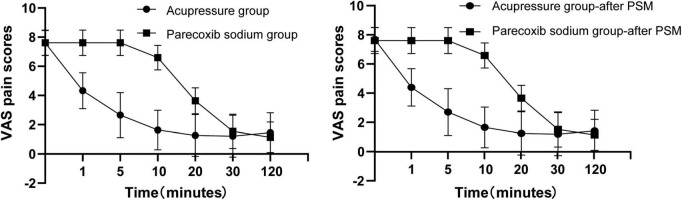
Visual analog scale (VAS) decrease between the acupressure group and parecoxib sodium group before and after propensity score matching (PSM).

As shown in Table 2, pre-treatment VAS scores were comparable between the acupressure and parecoxib sodium groups (*P* = 0.961). The median and IQR for the baseline of VAS in the acupressure and parecoxib sodium group were both 8, 1 (before PSM) and 8, 1 (after PSM). Both groups exhibited significant decreases in VAS scores before or after PSM. However, the acupressure group yielded lower VAS scores at the 1st, 5th, 10th, and 20th minute than the parecoxib sodium group (mean: 4.33 vs. 7.61, mean difference (MD): −3.28, 95% CI: −3.74, −2.84; mean: 2.65 vs. 7.61, MD: −4.96, 95% CI: –5.49, −4.44; mean: 1.63 vs. 6.59, MD: −4.96, 95% CI: −5.44, −4.48; mean: 1.26 vs. 3.64 MD: −2.38, 95% CI:−2.88, −1.87), *P* < 0.05 before PSM; (mean: 4.39 vs. 7.61, MD: −3.22, 95% CI: −3.70, −2.73; mean: 2.71 vs. 7.61, MD: −4.90, 95% CI: −5.47, −4.33; mean: 1.66 vs. 6.59, MD: −4.93, 95% CI: −5.43, −4.42; mean: 1.24 vs. 3.66 MD: −2.41, 95% CI: −2.95, −1.87) *P* < 0.05 after PSM. There was no significant difference in VAS score between the two groups at the 30th and 120th minute (Figure 3). The onset time (time taken to achieve a 50% VAS-score decrease relative to each patients’ VAS baseline) was remarkably different between the two groups (1.15 min of acupressure vs. 15.80 min of parecoxib sodium, MD: 14.64, 95% CI: 14.08, 15.21, *P* < 0.001) before PSM and (1.17 min of acupressure vs. 15.68 min of parecoxib sodium, MD: 14.51, 95% CI: 13.91, 15.11, *P* < 0.001) after PSM. These results indicate that acupressure achieved a faster remission of renal colic than parecoxib sodium within 20 min after administration. After the 20th minute, the groups exhibited the same efficacy. Furthermore, the recurrence rate within 24 h was almost the same (0.22 vs. 0.18 95% CI: −0.15, 0.21 before PSM, 0.24 vs. 0.20 95% CI: −0.15, 0.24 after PSM, *P* > 0.05). The markedly effective rate, which was defined as the proportion of patients achieving a >60% decrease within 30 min relative to each patient’ s baseline, was similar between the two groups (86.9% for acupressure vs. 88.6% for parecoxib sodium, 95% CI: −0.173, 0.143 before PSM, 87.8 vs. 87.8%, after PSM, 95% CI: −0.167, 0.167 Table 3). The adverse effects are shown in Table 4.

**TABLE 2:** Outcomes of each group.

	Before PSM								After PSM							
	**Acupressure group (*n* = 46)**		**Parecoxib sodium group (*n* = 44)**						**Acupressure group (*n* = 41)**		**Parecoxib sodium group (*n* = 41)**					
	**Mean**	**SD**	**Mean**	**SD**	**Mean difference**	**95% CI**		***P*^a^-value**	**Mean**	**SD**	**Mean**	**SD**	**Mean difference**	**95% CI**		***P*-value**
						**Lower limit**	**Upper limit**							**Lower limit**	**Upper limit**	
VAS before treatment	7.60	0.86	7.61	0.87	−0.01	−0.37	0.36	0.978	7.68	0.82	7.61	0.89	0.73	−0.30	0.45	0.700
1 min VAS	4.33	1.23	7.61	0.87	−3.28	−3.74	−2.84	0.000	4.39	1.28	7.61	0.89	−3.22	−3.70	−2.73	0.000
5 min VAS	2.65	1.54	7.61	0.87	−4.96	−5.49	−4.44	0.000	2.71	1.60	7.61	0.89	−4.90	−5.47	−4.33	0.000
10 min VAS	1.63	1.36	6.59	0.84	−4.96	−5.44	−4.48	0.000	1.66	1.39	6.59	0.87	−4.93	−5.43	−4.42	0.000
20 min VAS	1.26	1.44	3.64	0.89	−2.38	−2.88	−1.87	0.000	1.24	1.48	3.66	0.88	−2.41	−2.95	−1.87	0.000
30 min VAS	1.22	1.43	1.55	1.17	−0.33	−0.88	0.22	0.238	1.20	1.47	1.51	1.21	−0.32	−0.91	0.27	0.289
120 min VAS	1.46	1.36	1.14	1.05	0.32	−0.19	0.83	0.216	1.41	1.41	1.15	1.06	0.27	−0.28	0.82	0.334
Onset time (min)	1.15	0.36	15.80	1.89	14.64	14.08,	15.21	0.000	1.17	0.38	15.68	1.89	14.51	13.91,	15.11	0.000
Recurrence in 24 h	Number		Number		Proportion difference				Number		Number		Proportion difference			
Yes	10		8						10		8					
No	36		36					0.673	31		33					0.790
Proportion of Recurrence in 24 h	0.22		0.18		0.04	−0.15	0.21		0.24		0.20		0.04	−0.15	0.24	

^a^ Categorical variables were analysis by *chi-square* tests; Continuous variables were analysis by *t*-tests.

**TABLE 3:** Markedly effective rate in 30 min.

	Before PSM					After PSM				
	**Acupressure group (*n* = 46)**	**Parecoxib sodium group (*n* = 44)**	**Proportion difference**	**95% CI**		**Acupressure group (*n* = 41)**	**Parecoxib sodium group (*n* = 41)**	**Proportion difference**	**95% CI**	
				**Lower limit**	**Upper limit**				**Lower limit**	**Upper limit**
VAS decrease ≥ 60%	40	39				36	36			
VAS decrease < 60%	6	5				5	5			
Proportion	0.869	0.886	−0.017	−0.173	0.143	0.878	0.878	0.000	−0.167	0.167

Analysis by *chi-square* tests: *P* > 0.05.

**TABLE 4:** Adverse effects.

Adverse effects	Acupressure group (*n* = 46)	Parecoxib sodium group (*n* = 44)
Nausea and vomiting	0	0
Bloating	0	1
Narcolepsy	0	0
Dizziness	0	1
Rash	0	0
Itching/rash/bleeding at press point	0	0
Total	0	2

### 3.3. Linear mixed effects regression

Further linear mixed effects regression analysis was conducted to investigate the rate of decrease of VAS within 10 min between acupressure group and parecoxib sodium group and their covariant effect on the efficacy of acupressure. Covariant including age, sex, pain site (right or left), stone location, and stone size were enrolled in this model. Linear mixed effects regression was performed and the results are shown in Table 5. In this model, the estimate of intercept represented that the VAS score was 7.61 (95% CI: 6.75, 8.47, *p* = 0.000) under no any other factors. The acupressure group could significantly affect the VAS decreasing rate and gained an estimate of −2.05 (95% CI: −2.51, −1.59, *p* = 0.000) lower than the parecoxib Sodium group. Other covariant such as gender, age, pain site, stone location, and stone size showed no remarkable effect on VAS decreasing rate. The estimate of Time 1 represented the rate of change of VAS per minute among the controls before minute 10 and gained an estimate of −0.34 (95% CI: −0.39, −30, *p* = 0.000) which meant that is a decrease of 0.34 per minute. Time 2 represented the change of the before minute 10 rate of change of VAS per minute among the controls after minute 10 and gained an estimate of 0.31 (95% CI: 0.26, 0.36, *p* = 0.000). In another word, that is a decrease of 0.03 (−0.34 + 0.31) per minute past minute 10 among the controls. The interactive model found the estimate of Acupressure * Time 1 represented a difference in rate of change of VAS between the acupressure and the controls before minute 10 with an estimate of −0.15 (95% CI: −0.21, −0.08, *p* = 0.000), which indicated that acupressure is associated with a further 0.15 decrease per minute compared to the controls. The full rate of decrease for acupressure group before minute 10 is −0.15–0.34 = −0.49 per minute. However, parecoxib sodium group* Time 2 showed a better effect on VAS decreasing rate than acupressure group. The co-effect of them gained an estimate of 0.18 (95% CI: 0.12, 0.25, *p* = 0.000). It also meant the full rate of change among the acupressure group is 0.18–0.15 + 0.31–0.34 = 0.00 per minute after minute 10.

**TABLE 5:** The estimation of visual analog scale (VAS) decreasing rate in linear mixed effects models.

Parameters	Estimate	SE.	Df.	t	Significance	95% CI	
						**Lower limit**	**Upper limit**
Intercept	7.61	0.44	617	17.34	0.000	6.75	8.47
Acupressure^a^	–2.05	0.23	617	–8.75	0.000	–2.51	–1.59
Male^b^	0.26	0.13	617	1.96	0.051	0.00	0.52
Pain site: Left^c^	–0.20	0.13	617	–1.46	0.144	–0.46	0.07
Stone location: Renal pelvis and upper ureter	–0.25	0.14	617	–1.76	0.079	–0.53	0.03
Stone location: Middle ureter^d^	0.32	0.18	617	1.80	0.072	–0.03	0.67
Stone size: < 6 mm	0.01	0.22	617	0.04	0.968	–0.42	0.44
Stone size: 6 mm ≤ size < 10 mm^e^	0.12	0.15	617	0.85	0.398	–0.16	0.41
Age	0.01	0.01	617	1.20	0.230	–0.01	0.02
Time 1^f^	–0.34	0.24	617	–14.55	0.000	–0.39	–0.30
Time 2^g^	0.31	0.02	617	12.34	0.000	0.26	0.36
Acupressure * Time 1	–0.15	0.03	617	–4.38	0.000	–0.21	–0.08
Parecoxib sodium * Time 1	–	–	–	–	–	–	–
Acupressure * Time 2	0.18	0.03	617	5.29	0.000	0.12	0.25
Parecoxib sodium* Time 2	–	–	–	–	–	–	–

^a^ Compared with the parecoxib sodium group, the same below the interaction models.

^b^ Compared with female, the same below the interaction models.

^c^ Compared with the pain site: Right.

^d^ Compared with the renal pelvis, upper ureter and lower ureter.

^e^ Compared with the group of <6 mm and group of ≥10 mm, the same below the interaction models.

^f^ Time 1 was continuous variable equal to 0, 1, 5, 10, 20, 30, and 120 min.

^g^ Time 2 was continuous variable equal to 0, 0, 0, 0, 10, 20, and 110 min.

## 4. Discussion

With the widespread use of painkillers, including NSAIDs and opioids, side effects, such as nausea, vomiting, constipation, and drowsiness, have also received increasing attention. Thus, an increasing number of alternative therapies have been developed for acute renal colic. Treatment involving active warming of the abdomen and lower back region has been reported to be effective for renal colic (15). Acupuncture also plays an important role in renal colic, a phenomenon that has been proven in several clinical trials (16, 17). Acupressure, which is very similar to acupuncture, is an ancient Chinese treatment. It performs an analgesic function through the stimulation of acupoints using acute pressure, called acupressure. According to Chinese medicine, pressing these acupoints potentially regulates the balance of yin and yang through meridians (18). Current studies have demonstrated that neurotransmitters, such as serotonin, and the increase in pain threshold mediate the analgesic mechanism through the stimulation of acupoints (18–21).

Like other acupoints, the Qiu acupoint also exerts its effect through acute pressure. However, the Qiu acupoint is not the traditional acupoint that goes through the meridians; it is similar to the A-shi point, which reflects a specific pain area beyond the meridian acupoint, treated using alternative therapies, especially acupuncture and acupressure.

Our data demonstrated that acupressure at the Qiu acupoint achieves a similar effect to that of parecoxib sodium; excitingly, Qiu acupoint has a remarkably rapid effect. In our investigation, VAS scores decreased significantly in the 1st min in the acupressure group; however, an almost similar effect between the same groups was observed in the 30th min. This result is consistent with that obtained by Kaynars et al. (16), in which the acupuncture group experienced a drastic decrease after 10 min compared with the diclofenac and acetaminophen groups. Coincidentally, several studies have reported on analgesic treatment in locations near the Qiu acupoint. Gul and Gul (22) injected sterile water into the triangular area bound by the 12th costal margin, iliac crest, and vertebral spine for renal colic, and it had an effect similar to that of diclofenac sodium by muscle injection. Aydin et al. (23) performed a pilot clinical feasibility study and found that erector spinae plane block with 0.25% bupivacaine achieved a better effect than NSAIDs. The trigger point, which is described as a point located at an area bound by the costal margin, vertebral spine, and iliac crest, has been reported to be effective for renal colic by injecting lidocaine (24, 25). The Qiu acupoint lies near the middle of the triangular area bound by the 12th costal margin, iliac crest, and vertebral spine. This evidence reveals that a certain mechanism exists for analgesia in this triangular area. According to our study, acupressure at the Qiu acupoint appears easy to perform and does not require injection equipment or any medicine. In linear mixed effects regression analysis, we determined the rate of decrease of VAS within 10 min between acupressure group and parecoxib sodium group and their covariant effect on the efficacy of acupressure at the Qiu acupoint and found that acupressure had a better VAS decreased rate within 10 min compared to parecoxib sodium while there was no effect in other clinical factors including age, sex, pain site, stone location, and stone size. These result considered the co-effect of other variants and convinced the result that acupressure at the Qiu acupoint has a quicker Analgesic effect within 10 min compared to parecoxib sodium.

Acupressure has been considered a low-cost, low-risk, environmental- and patient-friendly treatment in China. More importantly, the alternative treatment potentially curtails the addictiveness of many analgesics, such as morphine, and side effects caused by NSAIDs. However, the worldwide promotion of acupressure still faces many challenges. First, there was a lack of high-quality evidence. In addition, the habit of using painkillers is not easy to alter. Thus, we hope our study provides some evidence regarding the utility of acupressure in treating acute renal colic.

Indeed, there are certain limitations to our study. First, the sample size was insufficient, and only a single center was used. We hope that a multi-center study or randomized controlled trials (RCTs) will be performed in the future to strengthen our conclusions. Second, bias was inevitably generated because of the nature of our cohort study compared with that of RCTs. Third, the investigation time of the protocol was limited to 30 min, which was based on the NSAID onset time. The duration of analgesia was also assessed at the 120th min. However, it might have been better to increase the detection time to the 60th and 90th minutes, thus rendering the study more complete.

## 5. Conclusion

In this study, we demonstrated that acupressure at the Qiu acupoint had a significant effect on reducing pain caused by renal colic, similar to that of parecoxib, but without significant adverse effects. Acupressure was significantly faster than parecoxib sodium in decreasing VAS scores within 10 min. Overall, the foregoing evidence presents an alternative therapy for acute renal colic.

## Data availability statement

The raw data supporting the conclusions of this article will be made available by the authors, without undue reservation.

## Ethics statement

The studies involving human participants were reviewed and approved by the Ethics Committee of the First Affiliated Hospital of Guangzhou University of Chinese Medicine (ethical approval number: No. ZYYECK【2020】083). Written informed consent for participation was not required for this study under the emergency clinic situation.

## Author contributions

HoL, YQ, and SX conceived and designed the study. CC and ZZ performed the study, collected and analyzed clinical data. CC wrote and revised the manuscript. ML, ZW, HaL, HM, JW, and MC participated in discussing the manuscript. All the authors approved the final version of the manuscript.
